# Herbal based nanoparticles as a possible and potential treatment of cancer: a review

**DOI:** 10.37349/etat.2025.1002285

**Published:** 2025-01-03

**Authors:** Roshan Yadav, Himmat Singh Chawra, Gaurav Dubey, Md Sabir Alam, Vikram Kumar, Pragya Sharma, Navneet Kumar Upadhayay, Tejpal Yadav

**Affiliations:** University of Valladolid, Spain; ^1^Department of Pharmaceutics, NIMS Institute of Pharmacy, NIMS University Rajasthan, Jaipur 303121, India; ^2^Department of Pharmacognosy, NIMS Institute of Pharmacy, NIMS University Rajasthan, Jaipur 303121, India; ^3^Department of Pharmaceutics, SGT College of Pharmacy, SGT University Haryana, Gurugram 122505, India; ^4^Amity Institute of Pharmacy, Amity University Rajasthan, Jaipur 303002, India

**Keywords:** Phytoconstituents, nanoparticles, cancer, nanotechnology, radiation therapy, chemotherapy, surgery, herbal

## Abstract

Cancer is the greatest cause of mortality worldwide. Various drug classes treat various cancers. Nanoformulations made from natural sources are being studied for treating several diseases, including cancer. Surgery, chemotherapy, immunotherapy, and radiation have mostly failed to treat cancer. These drugs may damage quickly dividing healthy tissues, structural anomalies, bodily toxicity, long-term side effects, tumor cell drug resistance, and psychiatric disturbances. Researchers are developing nanoscale medicines using natural medications like *Malva sylvestris* and *Curcumin* to lower concentrations and improve target specificity. Nanoparticles’ small size and unique properties make them beneficial. They encapsulate medicinal ingredients, improving solubility, medication release, cellular absorption, and delivery. Nanoparticles may better identify and bind to cancer cells when functionalized with ligands. Natural chemicals and nanotechnology may improve medication availability, distribution, and targeting to cancer cells, making cancer treatments more effective and safe. Nanomedicine, which employs nanoparticles to treat cancer and malignant cells, has grown rapidly because nanodrugs are more effective and have fewer side effects than current commercial cancer drugs. Nanotechnology-based natural chemicals and pharmaceutical delivery methods for cancer therapy are covered in this review article. The paper discusses nanoparticle pros and cons and natural chemicals’ cancer-fighting appeal.

## Graphical abstract

**Graphical abstract.**
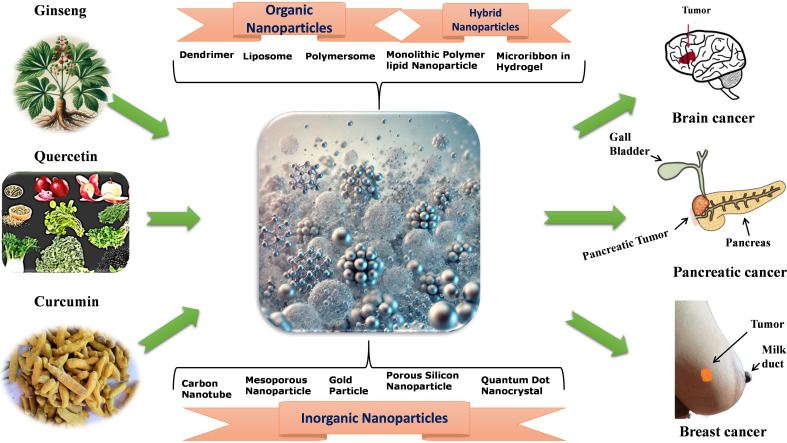
Herbal based nanoparticles for cancer treatment

## Introduction

Cancer is the second greatest cause of death worldwide, a significant public health concern, and one of the most common causes of illness and mortality overall [1]. The International Agency for Research on Cancer (IARC) has performed a comprehensive survey in 185 countries, including all age groups, sex categories, and 36 kinds of malignancies. The purpose of this study was to create a worldwide cancer burden database, which includes data on cancer incidences and cancer mortalities [2]. The data is sufficiently concerning to warrant a search for novel therapies that can surpass traditional treatments [3]. Cancer is caused by damage of genes which control the growth and division of cells. Genes carry the instructions for basic functions of cells [4]. Blood is needed for cancerous cells to proliferate. It is possible to treat cancer by eliminating it, interrupting the blood flow to the cells, or changing the genes that cause the damage verifying the cell development allows for detection and diagnosis. As such, the required instruments need to be very sensitive [5]. Despite several advancements in cancer therapy throughout history, the increasing number of instances highlights the need for a more profound comprehension and the development of novel therapeutic agents or the modification of current treatments to effectively manage the prevalence of the illness [2, 6]. Researchers and scientists are hoping to use nanotechnology to develop therapeutic compounds that target particular cells and release the toxin in a controlled, time-released [5, 7]. Nanotechnology is gaining global recognition as an essential component of biomedical research, specifically focused on cancer theranostics. Nano-formulations possess distinct characteristics such as a customizable surface and a significant surface area-to-volume ratio [8]. This technique enables the effective absorption and encapsulation of medicinal agents, such as phytochemicals, for targeted medication administration or passive distribution. In addition, these nanoformulations minimize the risk of causing harm to the whole body and improve the capacity of the drug to be absorbed and released at the intended location. Examples of carrier systems often used include liposomes, polymeric nanoparticles (NPs), and polymeric micelles [9]. Developing single agents with the dual capabilities of cancer detection and therapeutic delivery is the main goal. The NPs will travel throughout the body, identify molecular alterations linked to cancer, help with imaging, release a therapeutic substance, and then track how well the intervention is working [8, 10]. It is possible to diagnose, cure, and confirm the growth of the cells. The destructive mechanism of the genes can be corrected, the blood supply to the cells can be cut off, or the cells can be destroyed. X-rays, CT scans, and MRIs are used to observe the physical growth or changes in the organ [11]. A biopsy using cell culture is used to confirm the diagnosis of cancer [5]. Due to the fact that cells are only a few microns in size and NPs are few nanometers in size, NP can penetrate cells and access DNA genes, potentially enabling the detection of gene defects [12]. Radiation therapy, chemotherapy, and surgery are the standard cancer treatment choices. In nanotechnology, certain NPs can be engineered to selectively absorb specific wavelengths of radiation, which, if they penetrate malignant cells, will cause them to burn [13]. Therapeutic agents that target particular cells and deliver toxins to kill them can be created using nanotechnology [14].

Most people agree that cancer is a genetic disease that develops on its own cells and is caused by changes to the oncogene, tumor-suppressor, and genome-stability genes. But immunity, the stroma, and the tumor-cell microenvironment all play significant roles in cancer. Indeed, cancer cells must overcome both intrinsic (cell autonomous) and extrinsic (immune induced) hurdles to oncogenesis in order to progress to full-blown neoplasia. Tumor cells can only spread and ultimately destroy their host when they are able to subvert immune regulation [15, 16]. Consequently, the notion that the immune system influences the development of tumors in humans is supported by the higher incidence of certain solid tumors in immunocompromised patients, reports of spontaneous tumor regression, and the favorable prognostic effect of tumor-specific cytotoxic T lymphocytes (CTLs) or antibodies [17–19]. The NP will move throughout the body, identify molecular alterations linked to cancer, help with imaging, release a therapeutic substance, and then track how well the intervention is working [20]. In modern times, healthcare experts are seeking inspiration from traditional medicine to enhance the current treatment approaches by integrating herbal expertise with technology [21]. Herbal medicine, also known as phytomedicine, utilizes bioactive compounds obtained from plants/herbs to enhance overall health and wellness [22, 23]. Strong anticancer agents have historically been found in nature. These include the vinca alkaloids vincristine (VCR), vinblastine, vindesine, vinorelbine, taxanes paclitaxel (PTX), docetaxel, podophyllotoxin and its derivatives etoposide (ETP), teniposide, and a number of other medications that are derived from plants and that the US Food and Drug Administration (US FDA) has approved for use in cancer therapy [10]. Over thirty naturally occurring chemicals originating from plants have been found and are currently undergoing clinical trials. Additional plant-derived chemicals that are presently being studied include combretastatin A4, homoharringtonine, β-lapachone, and flavopiridol. Synthetic flavone flavopiridol is made from the plant alkaloid rohitukine, which was extracted from *Amoora rohituka*’s leaves and stems and then from *Dysoxylum binectariferum*. An inhibitor of cyclin-dependent kinase is flavopiridol [24–26]. This study focuses on novel plant-derived medicinal chemicals that have been shown in clinical studies to be effective in treating a variety of cancers [27].

## Phytoconstituents for the treatment of cancer

Presently, more than half of all anticancer medications licensed by the US FDA have natural origins, and more than 60% of all medications undergoing clinical trials for cancer have natural origins. Epidemiological research indicates that eating a diet high in phytochemicals, which includes fruits and vegetables, may lower one’s chance of developing cancer [11]. High concentrations of a wide variety of phytochemicals can be found in both fresh and processed fruits and food products. Polyphenols, which include anthocyanins and other flavonoids, hydrolysable tannins (ellagitannins and gallotannins), condensed tannins (proanthocyanidins), and other tannins, make up a significant component of these phytochemicals. Antioxidant is one of the proposed mechanisms by which polyphenols have anticancer effects [28].

One cannot undervalue the impact of natural ingredients on the development of anticancer drugs [29]. About 60% of all medications presently undergoing clinical trials for various cancers are either natural products, compounds derived from natural products, pharmacophores derived from active natural products, or “old medicines in new clothing”, indicating that natural compounds that have been altered have been connected to the targeting system [30]. We analyze almost 200 researches that looked at the connection between eating fruits and vegetables and malignancies of the breast, colon, lung, cervix, esophagus, stomach, bladder, pancreas, and ovary [31].

Human-consumed plants have thousands of phenolic chemicals in them. Due to dietary polyphenols’ potential anticarcinogenic and antioxidant properties, their effects are currently of great interest [6]. Dietary polyphenols are thought to be anticarcinogens since they are antioxidants, yet there isn’t enough concrete proof to support this theory. The inhibitory effects of phenolic acids and their derivatives, tea and catechins, isoflavones and soy preparations, quercetin (QC) and other flavonoids, resveratrol, and lignans on cancer are reviewed in this chapter along with the processes underlying them, based on investigations conducted in vitro and in vivo [32]. By altering the molecular processes at the beginning, promotion, and advancement phases of carcinogenesis, polyphenols may prevent it from occurring. Through their effects on estrogen-related activities, isoflavones and lignans may have an impact on the growth of tumors. Because the biological activity is determined by the tissue levels of the beneficial chemicals, there is a great deal of discussion over the bioavailability of dietary polyphenols [33]. Some recently used plants for cancer treatment summarized in Table 1.

**Table 1:** Comprehensive detail of some recently used plants for the treatment of cancer

**Name of plant with family**	**Plant part used**	**Formulation type**	**Cell line/Animal model**	**Type of cancer**	**Reference**
*Malva sylvestris* L. (Malvaceae)	Leaves	AgNPs	RCC-JW (KTCTL-195), RCC-GH, CaKi-2, HEK293 cell lines and HUVEC cell line	Renal cell carcinoma	[34]
*Hedra helix* (Araliaceae)	Leaves	AuNPs	MCF-7 and MDA-MB-231	Cytotoxic activities	[35]
*Senna alexandrina* (Fabaceae)					
*Thyme vulgaris* (Lamiaceae)					
*Tribulus terrestris* (Zygophyllaceae)					
*Mellissa officinalis* (Lamiaceae)	Leaf	Herbal NPs	HUVEC cell line	Human lung cancer	[36]
*Platycodon grandiflorum* [Jacq.] A. DC (Campanulaceae)	Dried radix and rhizome	Lipid-polymer NPs	Mice (BALB/c) 4T1 tumor-bearing mice	Breast cancer	[37]
*Curcuma zedoaria* [Berg.] Rosc (Zingiberaceae)					
*Curcuma longa* (Zingiberaceae)	Rhizome	AgNPs	HT-29 cell line	Human colon cancer	[38]
*Curcuma aromatic* (Zingiberaceae)					
*Curcuma caesia* (Zingiberaceae)					
*Rosmarinus officinalis* (Lamiaceae)	Leaves	Palladium NPs, platinum NPs, bimetallic (palladium + platinum) NPs	SW480 and LS180 cell line	Human colorectal cancer	[39]
Ginseng (Araliaceae)	Roots				
*Ammi visnaga* (Apiaceae)	Entire plant	AgNPs	HeLa cell line	Anticancer activity	[40]
*Artemisinin* (Asteraceae)		Niosomal NPs	SW480	Colorectal cancer	[41]
*Mangifera indica* (Anacardiaceae)	Peel	AuNPs	MDA-MB-231/female mice	Breast cancer	[42]
*Catharanthus roseus* (Apocynaceae)	Leaves	AgNPs	Human liver cancer (HepG2) cells	Hepatocellular carcinoma (HCC) or liver cancer	[43]
*Berberis thunbergii* (Berberidaceae)	Leaf	AgNPs	PANC-1, AsPC-1, and MIA PaCa-2	Human pancreatic cancer	[44]
*Salvia miltiorrhiza* (Lamiaceae)	Root	Lipid-polymer hybrid NPs	4T1 breast cancer tumor-bearing nude mouse model	Breast cancer	[45, 46]

AgNPs: Silver nanoparticles

### Phytoconstituents based NPs for cancer treatment

NPs are described as particles having a single dimension of less than 100 nm with special characteristics that are often absent from bulk samples of the same substance. NPs can be categorized as 0D, 1D, 2D, or 3D depending on their general shape [47]. The fundamental structure of NPs is composed of three layers: the surface layer, the shell layer, and the core, which is commonly referred to as the NP itself and is essentially the central section of the NP. This basic composition is highly complex. Due to their remarkable characteristics, such as high surface-to-volume ratio, dissimilarity, sub-micron size, and improved targeting mechanism, these materials have become increasingly significant in interdisciplinary sciences [48].

**Figure 1:**
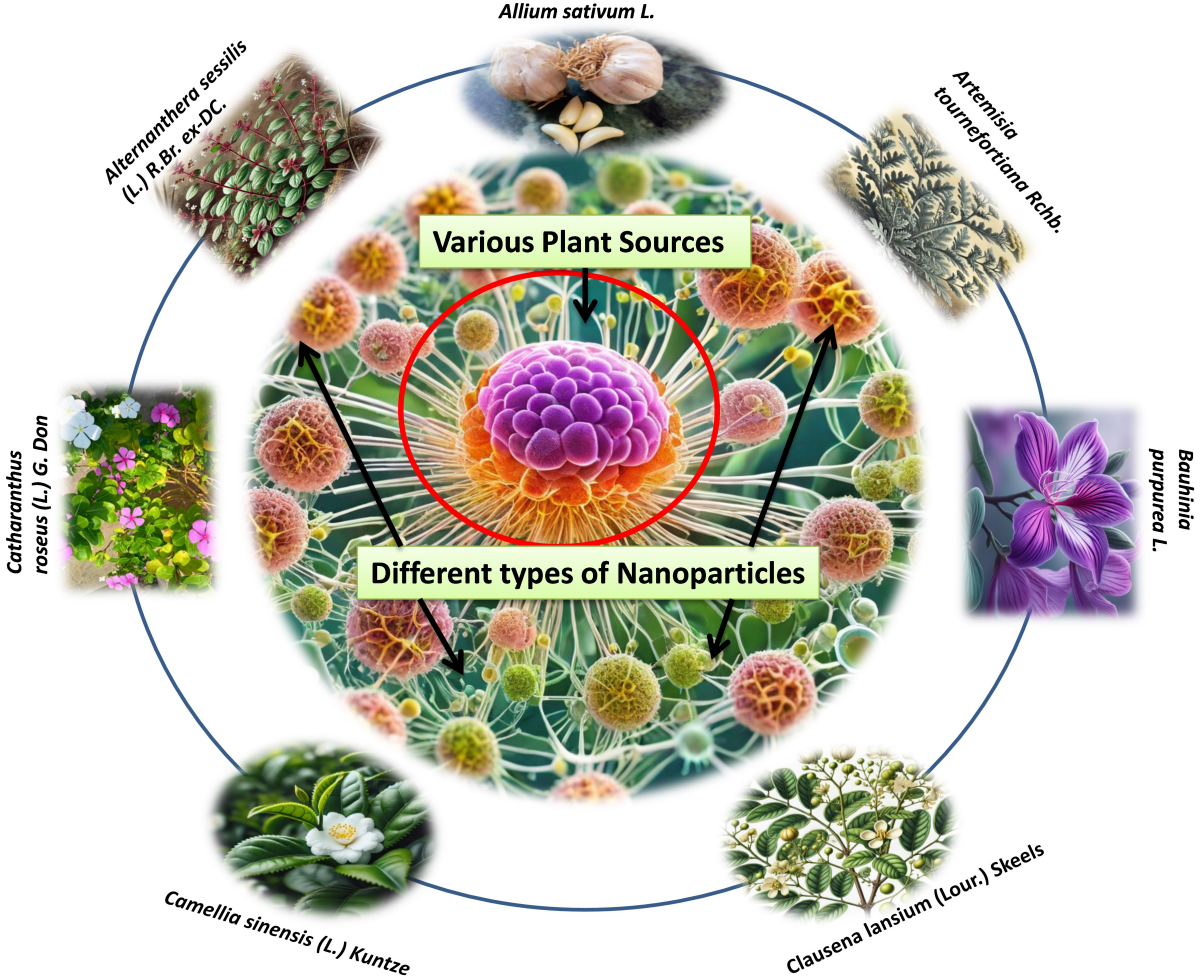
Different types of nanoparticles from various plant sources for the treatment of cancer

According to research, NPs can penetrate deep into tissues, increasing their permeability and retention capacity. Furthermore, the properties of the surface influence bioavailability and half-life by efficiently overcoming epithelial fenestration. As an illustration, NPs coated with the hydrophilic polymer polyethylene glycol (PEG) reduce opsonization and evade T cell clearance [49]. Furthermore, by adjusting the properties of particle polymers, it is feasible to maximize the rate of drug or active moiety release. In managing and treating cancer, the unique characteristics of NPs work together to control their therapeutic effect. In the last twenty years, a large number of therapies based on NPs have been released onto the market to help treat cancer [50]. New potential for the production of NPs for a variety of therapeutic applications have been made possible by developments in nanotechnology and a growing awareness of the significance of NP features (size, shape, and surface qualities) for biological interactions at the molecular level applications [51]. The field of cancer diagnosis and treatment could undergo a revolution thanks to nanotechnology. Because tumor angiogenesis is poorly regulated, a tumor is frequently linked with a faulty, leaky vascular architecture. An appropriately engineered nanoparticulate system that enables passive targeting and allows nanocarriers filled with cytotoxic chemicals to build up in the tumor tissues will benefit from this EPR phenomenon [52]. Drugs and drug delivery methods with modifications based on nanotechnology are being employed to treat cancer more and more frequently, with some even finding successful clinical applications. Improved cancer detection, more effective medication delivery to tumor cells, and molecularly tailored cancer therapy that enhances cancer patients’ therapeutic management is all possible with nanotechnology [53, 54]. Currently, a lot of researchers are more interested in plant-based medicine delivery that uses nanotechnology to reach the tumor location more deeply. Because of their improved solubility and hence bioavailability, site-specific targetability, decreased toxicities, and possible synergistic efficacy against various neoplasms, nanoparticulate systems present a viable platform for efficient phytoconstitutional administration [55]. Among various anticancer plants few plant based NPs are depicted in Figure 1.

### Type of NPs used with phytoconstituents for cancer treatment

#### Organic NPs

##### Polymeric NPs

It is generally known that polymeric NPs (PNPs) are “colloidal macromolecules” with a particular structural architecture made of several monomers [28]. Synthetic and natural polymers are utilized to prepare polymer NPs, which constitute a substantial class of drug delivery vehicles [48]. Polymer NPs are a versatile delivery system for a wide range of compounds, including as tiny chemicals, proteins, genes, and chemotherapeutic medicines. Poly (alkyl cyanoacrylate) (PACA), poly-caprolactone (PCL), polyanhydrides, polyethyleneimine (PEI), chitosan, gelatin, and polylactic acid (PLA) are just a few of the polymer NPs that are being studied in the lab. To accomplish controlled drug release in the target, the drug is either encapsulated or bonded to the exterior of NPs, forming a nanosphere or a nanocapsule [30, 56].

In recent *Ginsenoside* Rg5, a triterpene saponin derived from the herbal ginseng plant, has been shown recently to be among the most effective anticancer medications against several types of carcinoma cells. Rg5’s poor bioavailability, nontargeted administration, and low solubility in water, however, limit its therapeutic potential. In order to increase Rg5’s therapeutic efficacy and tumor targetability, researchers created folic acid (FA) modified bovine serum albumin (BSA) NPs (FA-Rg5-BSA NPs) [57].

For the oral delivery of QC, polymeric nanoparticulate systems from PLGA-TPGS (Qu-NPs) were used, and the anticancer impact of this formulation on TNBC was assessed both in vitro and in vivo. Qu-NPs have a good drug loading capacity (8.1 ± 0.4%) and a homogeneous spherical morphology, with a mean diameter of 198.4 ± 7.8 nm. Additionally, Qu-NPs showed noticeably better inhibition of TNBC cell growth and metastasis. After oral gavage, 4T1-bearing mice showed a strong anticancer impact of Qu-NPs with a tumor inhibition ratio of 67.88% and fewer lung metastatic colonies [58].

To combat breast cancer cells, Kumari et al. [59] created NPs of curcumin and PGMD (poly-glycerol-malic acid-dodecanedioic acid). NPs with an entrapment efficiency range of 75–81% were synthesized by combining two different ratios of PGMD polymer with curcumin: CUR NP 7:3 and CUR NP 6:4. The MCF 7 and MDA-MB-231 breast cancer lines were used for the scratch test and in vitro anticancer activities. At 48 hours, the CUR NP 7:3 and CUR NP 6:4 nanoformulations each had an IC50 of 40.2 μM and 33.6 μM, respectively, in the MCF-7 cell line; in the MDA-MB-231 cell line, the corresponding values were 43.4 μM and 30.5 μM. Assays using acridine orange/EtBr and DAPI labeling revealed apoptotic characteristics and nuclear abnormalities in the cells that were treated. Western blot study further supported this by showing that curcumin has a function in apoptosis via upregulation of caspase 9 [59].

##### Solid lipid NPs (SLN)

These are phospholipid monolayer, emulsifier, and water-based colloidal nanocarriers, with a size range of 1–100 nm. These are referred to as nanomaterials with zero dimensions. Triglycerides, fatty acids, waxes, steroids, and PEG are examples of the lipid component [31]. Lipids have been proposed as an alternate carrier to circumvent these drawbacks of polymeric NPs, especially for lipophilic drugs. SLNs are a class of lipid NPs that are gaining a lot of attention from formulators all over the world [32].

The cancer burden is rising rapidly worldwide, and it annually causes about 8.8 million deaths worldwide. Due to chemical drugs’ side effects and the emergence of resistance, the development of new green drugs has received much attention. We aimed to investigate whether solid-lipid NPs containing essential oil of *Zataria multiflora* (ZMSLN) enhanced the anticancer efficacy of the essential oil against breast cancer (MDA-MB-468) and melanoma (A-375) cells [60].

Studies on podophyllotoxin-based SLNs with epidermal targeting mechanisms have also been published. The findings showed that podophyllotoxin-based SLN preparations aided in drug penetration via the stratum corneum and hair follicle routes [61].

To target HT-29 cells for the therapy of colon cancer, the generated SLNs were further conjugated with FA. Even after coating chitosan on the surface of SLN, the optimization method yields the least amount of particle size (174 ± 5 nm). When compared to the uncoated formulation (25 μg mL^–1^), the chitosan-coated formulation shows greater cytotoxicity against HT-29 cells at a dosage of 10 μg mL^–1^. The medication is delivered by folate receptor-mediated endocytosis, which may be the cause of the increased cytotoxicity in FA conjugation. The techniques of western blot and fluorescence labeling were employed to verify the high-affinity binding of the folate receptor. The enhanced drug absorption and death in Ht29 cells are revisited with the aid of flow cytometry [3].

##### Liposomes

These are spherical vesicles that contain pharmacological molecules encapsulated in phospholipids, which can be either unilamellar or multilamellar [62]. Liposomes have distinctive properties, including minimal intrinsic toxicity, minimal immunogenicity, and biological inertness [63]. Following its description in 1965, the first closed bilayer phospholipid structures, known as liposomes, were quickly suggested as methods of delivering drugs. Significant technological advancements including remote drug loading, extrusion for homogeneous size, long-circulating (PEGylated) liposomes, triggered release liposomes, liposomes containing nucleic acid polymers, ligand-targeted liposomes, and liposomes containing drug combinations were made possible by the groundbreaking work of innumerable liposome researchers over the course of nearly five decades [64]. Because of their increased anti-tumor efficaciousness and improved absorption, liposomes offer a great vehicle for the administration of drugs including doxorubicin (Dox), PTX, and nucleic acid [65].

Liposomal-based phytochemical formulations have become more and more popular in recent years. Deshmukh et al. [66] employed chitosan and lecithin in an electrostatic deposition-assisted film hydration method to form a liposomal nanosystem that protected the flavone chrysin, also called 5,7-dihydroxyflavone, which is present in passion flowers, honey, propolis, *Passiflora caerulea* and *Passiflora incarnata*, and *Oroxylum indicum*, within the nanolipoidal shell [66].

This study investigates the effects of liposomal NP-delivered QC on the metabolism of mycophenolic acid (MPA) in combination therapy (impeding MPA metabolic rate). Both QC liposome NPs (QC-LNPs) and MPA liposome NPs (MPA-LNPs) were produced separately and thoroughly described. MPA-LNPs and QC-LNPs that were produced were measured to have sizes of 183 ± 13 and 157 ± 09.8, respectively [67].

##### Dendrimers

Spherical polymeric macromolecules with a well-defined hyperbranched topology are called dendrimers. Dendrimers are characterized by highly branching architectures [68]. Dendrimers typically have a size between 1 nm and 10 nm. Still, the size could be as much as 15 nm. A path to synthetic target molecules with spherical shapes, distinct surface chemistries, and sizes that correspond to virus particles is provided by the dendrimer chemistry described. The biggest aim is a generation 13 dendrimer made up of triazines connected by diamines, which is stable in the presence of additives and at different concentrations, pH levels, temperatures, and solvent polarity ranges [69].

A family of structurally defined macromolecules known as dendrimers has a central core, a high-density exterior that is terminated with surface functional groups, and a low-density inner made up of repeating branching units. Unlike their polymeric cousins, dendrimers are symmetrically structured and nanoscale particles that can be mass-produced in a reproducible manner using monodispersity technology [70].

For targeted applications, ursolic acid and FA were coupled with PAMAM dendrimer. The FA improves cellular absorption by targeting the folate receptor from HepG2 cells. The PAMAM dendrimer facilitates the cytotoxic action of ursolic acid and exhibits electrostatic absorptive qualities that assist attract HepG2 cells. PAMAM dendrimer is a potentially useful carrier for the targeted delivery of phytochemicals [71].

##### Nanoemulsions

Colloidal NPs with heterogeneous mixes of an oil droplet in aqueous media with a diameter ranging from 10 nm to 1,000 nm are known as nanoemulsions [40]. Advanced melanoma can be treated with a nanoemulsion of rapamycin, bevacizumab, and temozolomide [72]. In contrast to liposomes, nanoemulsions exhibit superior qualities, including stability, optical clarity, and biodegradability. In both cellular and animal models, the chosen medication combination loaded in IL shown encouraging results, most likely by influencing various mechanisms involved in tumor proliferation, dissemination, and angiogenesis. Future research would examine the impact of changing the chemical makeup of the nanoemulsion [73].

NEs have been used in the codelivery of several anticancer medications to increase the medications’ therapeutic efficacy and bioavailability. Mice with SKOV3 cancer were given a combination of PTX and curcumin in the form of a nanoemulsion by Ganta S and Amiji M. When PTX was given to mice treated with curcumin in nanoemulsion form, the AUC increased by 4.1 times. PTX’s relative bioavailability was 5.2 times higher, which enhanced the drug’s accumulation in cancer tissues by 3.2 times [74].

#### Inorganic NPs

##### Carbon NPs

As the name implies, carbon NPs are based on the element carbon. Since they are biocompatible and have optical, mechanical, and electrical qualities, they have been used extensively in the medical field [75]. The most promising options for various applications are the graphene family of nanomaterials because of their distinct intrinsic qualities, which are valued in their straightforward molecular design and their capacity to function in harmony with other nanomaterials already in existence [76]. Via differentiation-based nanotherapy, graphene oxide may be a useful non-toxic therapeutic approach for eliminating cancer stem cells [77]. A novel family of carbon compounds known as fullerenes (formerly buckminsterfullerenes) was first identified in 1985 [78]. If fullerene (Cm) is deposited in the tumor tissue, it should have a photodynamic effect on the tumor since it efficiently produces singlet oxygen when exposed to light [79].

**Figure 2:**
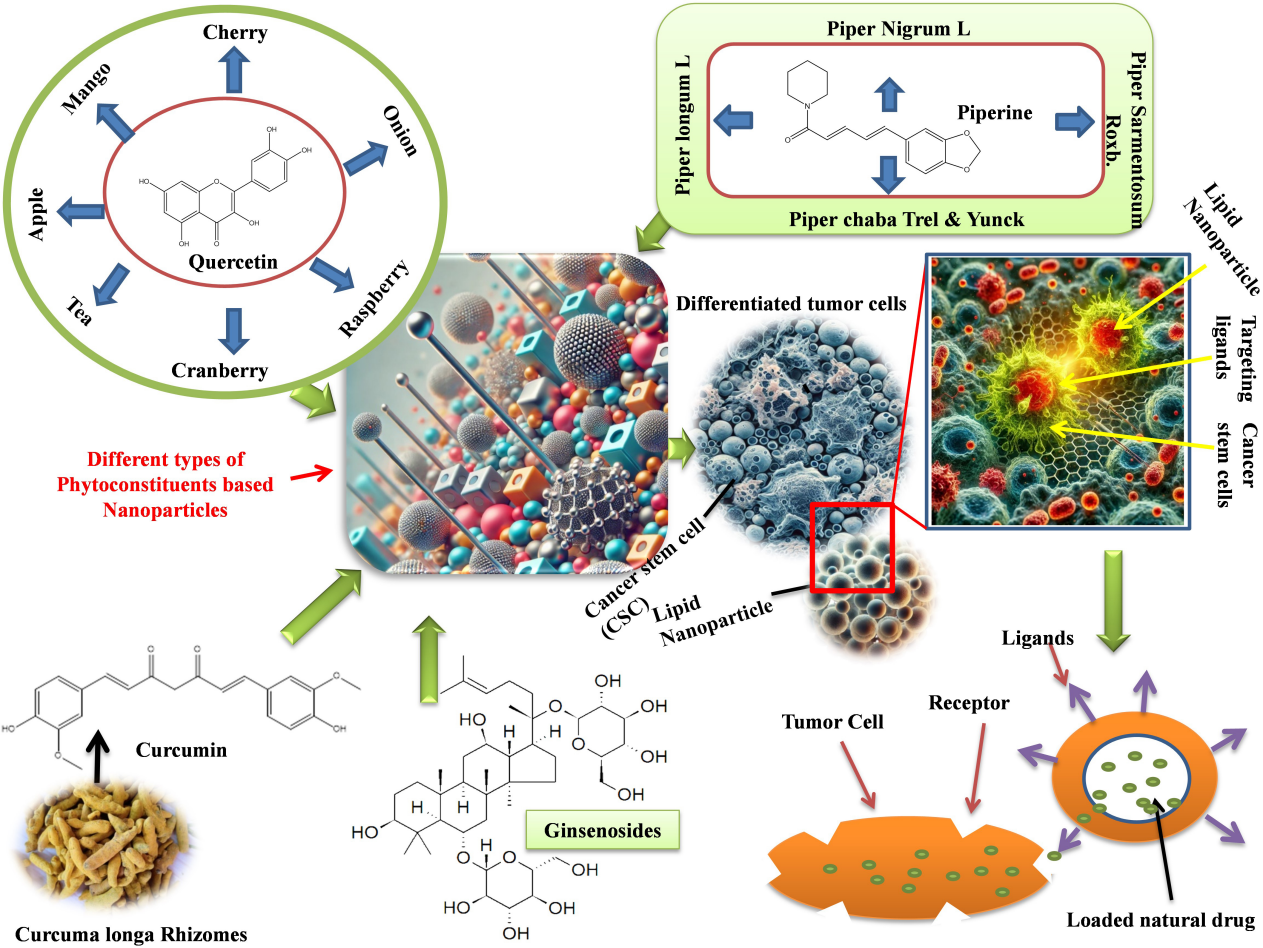
Mechanism of herbal nanoparticles to target the cancer stem cells and release of natural drug

Due to its low bioavailability, curcumin, a popular natural substance used in anticancer therapy, cannot reach its full potential. We further assessed the in vivo performance and in vitro properties of SWCNT-Cur, building on our earlier study of a new curcumin delivery system utilizing functionalized single-walled carbon nanotubes via phosphatidylcholine and polyvinylpyrrolidone (SWCNT-Cur). In mice, SWCNT-Cur dramatically raised the blood content of curcumin up to an eighteen-fold increase [78, 80]. Figure 2 depicted the mechanism of herbal NPs to target the cancer stem cells and release of natural drug.

##### Metallic NPs

Since metallic NPs have exceptional optical, magnetic, and photothermal capabilities, they are frequently investigated in “biological imaging” and targeted DDS. The most widely utilized metallic NPs include copper, silver, iron-based, and gold NPs. Because the size and surface characteristics of gold NPs are easily manipulated, they are exploited as intracellular targeted drug carriers [81]. The multidisciplinary topic of nanotechnology pertains to the engineering and design of items with a size less than 500 nanometers (nm). The National Cancer Institute has acknowledged that major advancements in cancer diagnosis and therapy can be achieved using nanotechnology, which presents an amazing and transformative opportunity. Nanotechnology has been researched and developed during the past few decades, mostly for application in cutting-edge medicine delivery systems [82]. Most cancer-related deaths are caused by metastases. Treatment of metastases presents distinct challenges because to their tiny size, high multiplicity, and dispersion into various organ settings [83].

Another naturally occurring alkaloid that is derived from a variety of plants, including *Canzptotheca acirminata* and *Mappia foetida*, is camptothecin. By specifically targeting topoisomerase I, an enzyme involved in the relaxation of DNA supercoils, this drug exhibits strong antitumor efficaciousness [84].

In Asia, ginseng (*Panax ginseng*) root is a common traditional medication. Ginseng consumption prior to a cancer diagnosis raised the overall survival rate for patients with breast cancer. Consuming *P. ginseng* improved several elements of the physical and mental functioning of patients with gynecologic or hepatobiliary cancer in another randomized placebo-controlled experiment. Furthermore, *European mistletoe*, or *Viscum album*, is another often recommended cancer treatment. Up to 2003, this plant extract was the subject of about 23 clinical investigations, 19 of which produced encouraging findings regarding the quality of life, survival, and tumor suppression of cancer patients [53].

##### Quantum dots

Semiconductor quantum dots (QDs) are light-emitting, nanoscale particles with special optical and electronic characteristics. These include the capacity to simultaneously excite multiple fluorescent colors, improved signal brightness, and stability of the fluorescent signal [68]. In cultivated HeLa cells, quantum dots labeled with the protein transferrin underwent receptor-mediated endocytosis, while dots labeled with immunomolecules identified certain antibodies or antigens [85]. The features of semiconducting quantum dots are highly uncommon; these particles are in the nanometer range. Band gaps in the quantum dots depend intricately on several aspects that are outlined in the article [86]. A new family of inorganic fluorophores known as QDs is becoming more well-known due to its remarkable photophysical characteristics [87]. A targeted cancer imaging, treatment, and sensing system utilizing QD-aptamer (Apt)-Dox conjugate [QD-Apt(Dox)] [88].

C-dots (aqueous fluorescent) of turmeric, black pepper, cinnamon, and red chili were made using a one-pot green synthesis process and subsequently subjected to an in vitro analysis. Human kidney cells (HK-2) and human glioblastoma cells (LN-229) were revealed to be more cytotoxic in cytotoxicity assays [89].

##### Magnetic NPs

MRI imaging often uses magnetic NPs, and medication delivery involves metal or metal oxides [90]. Lipid-based gene transfection techniques and magnetic NPs were used to induce active Fas expression in breast cancer cells. Human Fas and GFP-expressing plasmid DNA (pDNA) was transfected into MCF-7 breast cancer cells [91]. When the tissues were injected with LHRH-SPIONs, the contrast enhancement of conventional T2 images acquired from the tumor tissue and of mice bearing breast cancer xenograft is demonstrated to be significantly greater than that in saline controls [92]. Breast cancer xenografts and lung metastases were also observed to have improved MRI contrast in magnetic anisotropy multi-CRAZED images of tissues taken from animals treated with SPIONs [93, 94]. For the targeted treatment of oral squamous cell carcinoma, combining thermal ablation with antibody-targeting magnetic NPs is a viable treatment option [95].

The curcumine-loaded folate-grafted magnetic NPs have strong inhibitory effects on MCF-7 breast cancer cells and KB nasopharyngeal carcinoma cells. The NPs demonstrated targeted thermo-chemotherapy resulting in apoptosis by selectively interacting with the folate receptor, which is overexpressed in cancer cells, according to the magnetic effect [96].

#### Hybrid NPs

In order to overcome the limitations of single-component NPs, improve properties, achieve new properties not achievable for single NPs, and/or achieve multiple functionalities for single NPs, hybrid NPs are constructed from at least two different NPs. Various hybrid nanostructures, include Janus, dot-in-nanotube, dot-on-nanorod, heterodimer, core-shell, yolk-shell, and nanobranches [97]. There are four different types of hybrid nanomaterials: mesoporous silica, gold, or iron oxide NPs combined with biodegradable polymers or biomacromolecuels to form inorganic NP/organic polymer composite systems; polysilsesquioxane (PSQ) NPs synthesized from condensation of silanol-based monomers; and nanoscale coordination polymers (NCPs) and nanoscale metal-organic frameworks (NMOFs) composed of metal ions or clusters connected by organic linkers [98].

Hybrid NPs based on two design methodologies (tanker vs. barge), where a NP’s surface is coated with a nanotube system or contains liposomal, micellar, porous silica, polymeric, viral, noble metal, and nanotube systems [99–101]. Scientists draw attention to the design elements that must be taken into account to produce efficient nanodevices for the diagnosis and treatment of cancer [63, 64].

In recent curcuma longa is a plant and it has been utilized in several civilizations to treat a variety of illnesses. Its poor oral absorption, fast metabolism, low solubility in water and plasma, and chemical instability in alkaline environments limit its utilization. Furthermore, numerous plant species produce the phytoalexin Res (3, 4’, 5-trans-trihydroxystilbene), a polyphenol with a wide range of biological characteristics that have been thoroughly investigated in vitro and in vivo. Regretfully, Res exhibits low bioavailability, poor chemical stability, and low water solubility, all of which restrict its therapeutic efficacy. Cur likewise shares these characteristics. Because Cur and Res can serve as multifunctional agents, they are both claimed to have considerable anticancer activities [102].

#### Exosomes

In recent years, there has been a rise in documents highlighting the significance of exosomes (EXOs) in cancer biology [103]. Researchers are actively studying plant-derived EXO-like NPs (PENs) as a potential alternative to EXOs produced by mammalian cells. This allows them to overcome the technical limitations associated with mammalian vesicles. Polymer-based NPs (PENs) have great potential as nanocarriers in drug delivery systems due to their physiological, chemical, and biological characteristics. They are particularly effective in delivering different doses of drugs, especially in situations that need large-scale repeatability [104]. EXOs, the tiniest extracellular vesicles present in bodily fluids, serve as carriers of biomolecules, facilitating the passage of information between cells. This attribute makes them valuable as conveyors. Due to the similarity between the membranes of these NPs and cell membranes, they may be readily transported to convey various components. Due to their limited solubility in liquid, EXOs may be a viable method for loading chemotherapeutic medicines. This cancer therapy has the potential to eliminate the need for administering large amounts of medications via injections and instead offers a more suitable method of drug release [103].

Researchers have devised a novel approach to selectively induce programmed cell death in breast cancers and prevent their spread to the lungs. They achieved this by using natural nanovehicles derived from tea flowers, known as TFENs. The nanovehicles exhibited particle sizes of 131 nm, shape resembling EXOs, and negative zeta potentials. Cell tests demonstrated that TFENs had potent lethal effects on cancer cells by inducing the proliferation of reactive oxygen species (ROS). Elevated levels of intracellular ROS not only induce mitochondrial damage, but also halt the cell cycle, leading to the inhibition of cell proliferation, migration, and invasion in breast cancer cells in laboratory settings. Subsequent studies on mice revealed that TFENs, whether administered intravenously or orally, may concentrate in breast tumors and lung metastatic sites. They have the ability to hinder the development and spread of breast cancer and also influence the composition of gut microbiota [105, 106].

Crab haemolymph, as studied by Rezakhani et al. [107], was shown to contain EXOs that are rich in proteins and had antioxidant properties. These EXOs were seen to have the potential to exert anti-cancer effects on 4T1 cells. These EXOs might be suggested for breast cancer therapies [108].

Marine algae produce a diverse range of metabolites with various biological functions, and several studies have shown their ability to inhibit the growth of cancer cells. Multiple research has documented the anti-cancer properties of algae. It has been found that the EXOs generated from algae may have a suppressive impact on the proliferation of cancer cells [107].

Timely identification of cancer may significantly enhance its management. Recent research in the field of cancer detection and treatment has moved its attention to EXO biomarkers, which consist of a variety of RNA and proteins. In order to detect malignant activity at an early stage, microRNAs (miRNAs) may be isolated from cancerous cells present in the circulatory system’s extracellular vesicles (EXOs). Distinct markers may be used to identify EXOs originating from cancer that include miRNAs, which might potentially provide a more dependable and accurate method for early diagnosis [109]. The anticancer activities of various phytoconstituents are comprised in Table 2.

**Table 2:** Anticancer activity of different phytoconstituents

**S. No.**	**Active constituents**	**Activity**	**Animal model**	**Major finding**	**References**
1.	Ginsenosides	Antitumor	MCF-7 xenograft mouse model	FA-Rg5-BSA NPs had more efficacy in suppressing tumor development compared to Rg5 and Rg5-BSA NPs, demonstrating improved tumor accumulation capability.	[57]
2.	QC, miquelianin, isoquercetin	Antitumor and antimetastatic efficacy	Orthotopic-4T1 breast tumor model	Qu-NPs enhanced the notable anticancer and antimetastatic activities by suppressing uPA.	[58]
3.	Tymol, carvacrol, linalool, p-cymene	Antitumor	Homogenous matrix model	ZMSLN exhibited much superior antitumor efficacy compared to ZMEO and ZMSLN had a clear inhibitory impact on cell proliferation.	[60, 110]
4.	Poly-*d*-glucosamine	Antimetastatic and antitumor	Hepatocarcino-ma cells model	Increased cell-killing power and improved ability to be absorbed by the body.	[66, 111]
5.	Curcumin	Antitumor, brain tumors, breast and pancreatic cancers	Xenograft mouse model	FA-functionalized NPs with a magnetic field improved the cytotoxicity of drug-loaded nanocomposites.	[74, 112]
6.	Campthothecin	Antitumor	Albumin-bound PTX model, tumor xenografts	A more easy administration and efficacy and safety data support nab-PTX as a breast cancer reference taxane.	[84, 113]
7.	Piperine	Antitumor and prostate cancer	Nude mice model xenografted	Black pepper has been identified as a powerful nutraceutical that may effectively inhibit the development of chronic myeloid leukemia.	[89, 114, 115]

BSA: bovine serum albumin; FA: folic acid; NPs: nanoparticles; PTX: paclitaxel; QC: quercetin

## Challenges in conventional chemotherapy with phytochemicals

Phytochemicals, despite their significant potential as anticancer medications, face several limitations with conventional system of delivery. These include low solubility, poor bioavailability, high dosage requirements, a narrow therapeutic range, rapid absorption by healthy cells, a large apparent volume of distribution resulting in drug accumulation in normal cells, high clearance rate, and a short elimination half-life [4, 116, 117].

## Advantages of NPs in cancer therapy

A new era in cancer diagnosis, therapy, and management has been ushered in by the application of nanotechnology. NPs increase the intracellular concentration of medications while avoiding harm in healthy tissue by active or passive targeting [65]. One major concern for tumor therapy, particularly photodynamic therapy (PDT), has been targeted medication delivery. Researches goal is to improve photosensitizer (PS) targeting efficiency at the tumor site in vivo by employing folate-modified NPs [118].

Several studies have demonstrated that the trapping of anticancer medicines in submicronic colloidal systems (NPs) can influence their distribution profiles in both tissues and cells. The goal of this strategy is to lessen systemic adverse effects while increasing antitumor efficacy [119]. The NPs are divided into three categories: (i) magnetite NPs; (ii) various kinds of inorganic material-based NPs that are typically utilized for medication delivery, gene therapy, and other applications; and (iii) organic material-based NPs [120]. The advantage of using NPs to target cancer is that they can do so passively, by simply building up and being lodged in tumors [121]. The enhanced permeation and retention effect, which is brought on by leaky angiogenetic arteries and inadequate lymphatic drainage, has been used to explain why tumors have higher ratios of macromolecules and NPs than normal tissues [122].

## Clinical studies and patents of herbal medicines in cancer treatment

Clinical studies play a pivotal role in assessing the safety, efficacy, and mechanism of herbal medicines in cancer care. These studies range from preclinical research to advanced clinical trials.

### Preclinical studies

Focus on identifying bioactive compounds in herbs that exhibit anti-cancer properties. Common examples include flavonoids, alkaloids, saponins, and terpenoids. Laboratory studies on cell lines and animal models have shown that certain herbs, such as *Curcuma longa* (turmeric) and *Camellia sinensis* (green tea), can inhibit cancer cell proliferation, induce apoptosis, and prevent angiogenesis [123].

### Clinical trials

Herbal extracts like *Taxus brevifolia* (source of PTX) and *Catharanthus roseus* (source of vincristine and vinblastine) have undergone rigorous clinical trials and are now components of mainstream chemotherapy [124].

Recently, herbs such as *Withania somnifera* (ashwagandha), *P. ginseng* (ginseng), and *Tinospora cordifolia* (guduchi) are being studied for their role in immunomodulation and reducing chemotherapy-induced side effects [125, 126]. Some recent clinical studies are listed in Table 3.

**Table 3:** Recent clinical studies for cancer treatment by herbal medicines

**NCT number**	**Study phase with sponsor name**	**Study title**	**Type of condition**	**Treatment**	**Reference**
NCT05897749	Phase 4, Guang’anmen Hospital of China Academy of Chinese Medical Sciences	Clinical Study on the Effect of Brucea Javanica Oil Emulsion Injection on the Survival of Patients With Advanced Colorectal Cancer Who Failed to Receive Multi-line Treatment	Colorectal cancer	Brucea javanica oil emulsion injection	[127]
NCT03986528	Phase 4, Jie Li	Clinical Trial on the Survival Advantage of Kanglaite Injection (KLTi) in Advanced Non-Small Cell Lung Cancer (NSCLC)	Non-small cell lung cancer (NSCLC)	Kanglaite injection + chemotherapy	[128]
NCT05229809	Phase 4, Jie Li	Yiqi Wenyang Jiedu Prescription in the Prevention and Treatment of Postoperative Metastasis and Recurrence of Gastric Cancer: A Randomized, Double-blind, Controlled and Multi-center Clinical Study	Gastric carcinoma	Yiqi Wenyang Jiedu prescription	[129]
NCT01142479	Phase 2, phase 3, Taipei City Hospital	The Effects of Compound Herbal Formula (TPE-1) for Leukopenia and Cancer-related Fatigue in Breast Cancer Patients With Radiotherapy	Breast cancer	Chinese herbal medicine decoction	[130, 131]
NCT04403529	Phase 3, Fudan University	Evaluating the Clinical Value of Traditional Chinese Medicine in the Adjuvant Therapy of Triple-negative Breast Cancer	Triple negative breast cancer	Traditional Chinese medicine formulation	[132]
NCT03716518	Phase 3, Xiyuan Hospital of China Academy of Chinese Medical Sciences	Effect of TCM-TSKSR on Completion Rates of Chemotherapy in Patients With Stage II & III Colon Cancer: A Randomized Placebo-Controlled Clinical Trial	Colon cancer	Tonifying spleen and kidney sequential regimen	[133]
NCT04546607	Phase 2, phase 3, Natureceuticals Sdn Bhd	A Multicenter, Randomized, Double-Blind, Placebo-Controlled, Parallel, Study Assessing NuvastaticTM (C5OSEW5050ESA) 1,000 mg (3 Times a Day) in Improving Fatigue in Patients With Solid Stage I–IV Tumors	Astheni, cancer	Nuvastatic TM (C5OSEW5050ESA) 1,000 mg	[134, 135]
NCT03384667	Phase 2, phase 3, Seong-Gyu Ko	Efficacy and Safety Evaluation of Maekmoondong-tang on Post-operative Cough in Patients With Lung Cancer -Randomized, Double-blind, Placebo-controlled Clinical Trial	Lung cancer	Maekmoondong-tang	[136, 137]
NCT01441752	Phase 3, xuling	State Administration of Traditional Chinese Medicine of Shanghai	Non-small cell lung cancer	TCM	[138, 139]
NCT02929693	Phase 3	Clinical Study of Yiqi-yangyin-jiedu Decoction Combined With Gefitinib in Advanced Pulmonary Adenocarcinoma Patients With Activating EGFR Mutation	Cancer	Yiqi-yangyin-jiedu decoction	[140]

### Patents in herbal medicine for cancer

Patenting herbal formulations is essential for translating research into therapeutic applications. It protects intellectual property, incentivizes innovation, and facilitates commercialization.

#### Patenting trends

The number of patents related to herbal anti-cancer drugs has increased, particularly in China, India, and the United States. Examples include patents for herbal combinations targeting specific cancers, novel methods of herbal compound extraction, and formulations that enhance bioavailability or reduce toxicity [141, 142].

#### Key patented compounds and innovations

##### Artemisinin

Derived from *Artemisia annua*, it is patented for its anti-cancer and anti-inflammatory properties [143].

##### Curcumin derivatives

Patents for curcumin analogs that have improved solubility and efficacy against various cancers [144, 145].

##### Polysaccharides

Extracted from herbs like *Ganoderma lucidum* (Reishi mushroom) and patented for their immunomodulatory and anti-cancer properties [146, 147].

## Phytochemistry of chemical constituents loaded into NPs displaying anticancer activity

**Figure 3:**
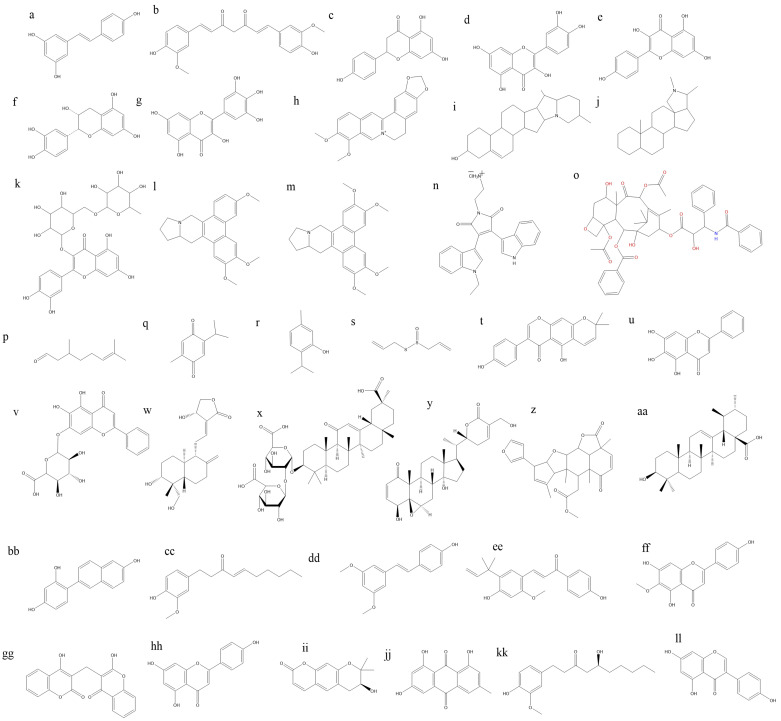
**Chemical structures of some anticancer phytoconstituents**. a: resveratrol; b: curcumin; c: naringnin; d: quercetin; e: kaepferol; f: epicatechin; g: myricetin; h: berberine; i: solaidine; j: conanine; k: rutine; l: antoline; m: tylophorine; n: BMA-155Cl; o: paclitaxel; p: citronellal; q: thymoquinone; r: thymol; s: allicin; t: alpinumisoflavone; u: baicalein; v: baicalin; w: andrographolide; x: glycyrrihizin; y: withaferin A; z: nimbolide; aa: ursolic acid; bb: resveratrol analogue HS-1793; cc: 6-shogaol; dd: pterostillben; ee: licochalcone A; ff: hispudilin; gg: dicoumarol; hh: apigenin; ii: decursinol; jj: emodin; kk: gingerol; ll: genistein

Phytoconstituents, also known as phytochemicals, are naturally occurring compounds found in plants that exhibit various biological activities, including anticancer properties [148, 149]. As shown in Figure 3 numerous phytoconstituents have been identified and studied for their potential to prevent or treat cancer. Many other plant-derived compounds are being studied for their potential as anticancer agents, and ongoing research continues to explore their mechanisms of action and therapeutic applications in cancer prevention and treatment [150]. Here are some examples of phytoconstituents with anticancer activity.

### Curcumin

Chemically, curcumin is a polyphenolic compound belonging to a class of compounds called curcuminoids. Its chemical structure consists of two aromatic rings linked by a seven-carbon chain with several functional groups [151].

The structure of curcumin plays a crucial role in its anticancer activity. Several structural features contribute to its ability to inhibit cancer cell growth and induce apoptosis.

#### β-Diketone structure

The central β-diketone structure of curcumin is essential for its anticancer activity. This structure is responsible for curcumin’s ability to chelate metal ions, particularly iron and copper, which are involved in various cellular processes. By chelating these metal ions, curcumin can disrupt metal dependent enzymes and signaling pathways implicated in cancer cell proliferation and survival [152].

#### Aromatic rings and hydrophobicity

The presence of aromatic rings in curcumin enhances its hydrophobicity, allowing it to interact with hydrophobic regions of proteins and cell membranes. This property enables curcumin to penetrate cancer cells and target intracellular signaling pathways involved in cell growth, apoptosis, and angiogenesis [153].

#### Methoxy and hydroxyl groups

The methoxy (-OCH3) and hydroxyl (-OH) groups attached to the aromatic rings of curcumin contribute to its antioxidant and anti-inflammatory properties. These functional groups scavenge free radicals and inhibit oxidative stress, which can promote DNA damage and cancer development. Additionally, they modulate inflammatory signaling pathways implicated in cancer progression [153].

#### Enol form

The enol form of curcumin, which predominates under physiological conditions, is believed to be the biologically active form responsible for many of its pharmacological effects, including its anticancer activity. The enol form participates in various molecular interactions and enzymatic reactions involved in cancer cell growth inhibition and apoptosis induction.

#### Conjugated system

Curcumin’s conjugated system, formed by alternating single and double bonds within its structure, contributes to its stability and ability to act as an electron donor or acceptor in redox reactions. This property enables curcumin to modulate intracellular redox balance and interfere with redoxsensitive signaling pathways implicated in cancer development and progression [152, 154].

Overall, the structural features of curcumin, including its β-diketone motif, aromatic rings, hydrophobicity, functional groups, enol form, and conjugated system, collectively contribute to its anticancer activity by targeting multiple molecular targets and signaling pathways involved in cancer pathogenesis. Understanding the structure-activity relationship of curcumin is essential for the rational design of novel curcumin derivatives with improved efficacy, bioavailability, and specificity for cancer therapy.

### Ginsenosides

The structure of ginsenosides is complex and consists of a steroid backbone with attached sugar moieties [155]. Several structural features contribute to the pharmacological activities of ginsenosides, including their anticancer properties.

#### Triterpene structure

Ginsenosides have a triterpene backbone composed of four fused rings, which is essential for their biological activity. This structure is responsible for the ability of ginsenosides to interact with cellular membranes and modulate signaling pathways involved in cancer cell growth and survival.

#### Sugar moieties

Ginsenosides contain one or more sugar moieties attached to the triterpene backbone, such as glucose, xylose, or arabinose. These sugar residues can affect the pharmacokinetics and bioavailability of ginsenosides and may influence their interactions with cellular targets [156].

#### Aglycone and glycoside forms

Ginsenosides exist in both aglycone (non-sugar) and glycoside (sugar-bound) forms, with different pharmacological properties. Aglycone ginsenosides, such as protopanaxadiol (PPD) and protopanaxatriol (PPT), are more readily absorbed and metabolized in the body compared to glycoside forms.

#### Hydroxyl groups

Ginsenosides contain hydroxyl (-OH) groups at various positions on the triterpene backbone. These hydroxyl groups can influence the biological activity of ginsenosides by affecting their solubility, stability, and interactions with cellular targets [156, 157].

Overall, the structural features of ginsenosides, including their triterpene backbone, sugar moieties, hydroxyl groups, collectively contribute to their anticancer activity. Understanding the structure-activity relationship of ginsenosides is essential for optimizing their therapeutic potential and developing novel anticancer agents derived from *P. ginseng*.

### Quercetin

QC belongs to the flavonoid group of polyphenolic compounds. Its chemical structure consists of three rings (A, B, and C) with multiple hydroxyl (-OH) groups attached. The presence of these hydroxyl groups contributes to its antioxidant activity, which helps neutralize harmful free radicals and reduce oxidative stress in cells. This oxidative stress is often associated with cancer development [158].

#### Antioxidant activity

QC’s potent antioxidant properties make it effective in protecting cells from oxidative damage, which can lead to mutations and cancer initiation. By scavenging free radicals and inhibiting oxidative stress, QC helps maintain cellular integrity and reduce the risk of carcinogenesis.

#### Anti-inflammatory effects

Chronic inflammation is closely linked to cancer development and progression. QC exhibits strong anti-inflammatory effects by inhibiting the production of inflammatory mediators such as cytokines and prostaglandins. By reducing inflammation, QC may help create an environment less conducive to cancer growth.

#### Apoptosis induction

QC has been shown to induce apoptosis, or programmed cell death, in cancer cells. This process is essential for removing damaged or abnormal cells from the body and preventing tumor growth. QC activates signaling pathways that trigger apoptosis in cancer cells, leading to their death.

#### Inhibition of angiogenesis

QC can inhibit the formation of new blood vessels (angiogenesis) that supply nutrients and oxygen to tumors, thereby slowing down tumor growth and metastasis. By disrupting the angiogenic process, QC helps deprive cancer cells of essential resources for their survival and proliferation.

#### Modulation of cell signaling

QC can modulate various cellular signaling pathways involved in cancer progression, including those related to cell proliferation, survival, and metastasis. By interfering with these signaling pathways, QC exerts control over cancer cell behavior and inhibits tumor growth and spread.

#### Chemopreventive effects

Numerous studies suggest that QC may have chemopreventive properties, meaning it can help prevent the initiation and progression of cancer. Its antioxidant, anti-inflammatory, and pro-apoptotic activities collectively contribute to its ability to protect against carcinogenesis.

In conclusion, the multifaceted properties of QC, including its antioxidant, anti-inflammatory, pro-apoptotic, anti-angiogenic, and signaling modulation effects, make it a promising natural compound for cancer prevention and therapy. However, further research, including clinical trials, is needed to fully elucidate its efficacy, safety, and optimal dosing regimens in cancer management [159, 160].

### Carvacrol

It is a natural monoterpenic phenol found in various essential oils, including oregano oil and thyme oil. It possesses a wide range of pharmacological properties, including antimicrobial, antioxidant, anti-inflammatory, and anticancer activities. While much of the research on carvacrol has focused on its antimicrobial effects, emerging studies suggest its potential as an anticancer agent [161]. Here’s how carvacrol’s properties relate to its anticancer activity.

#### Induction of apoptosis

Carvacrol has been shown to induce apoptosis, or programmed cell death, in cancer cells through various mechanisms. It activates signaling pathways that lead to apoptosis, including the mitochondrial apoptotic pathway and the death receptor pathway. By triggering apoptosis in cancer cells, carvacrol can effectively inhibit tumor growth and progression.

#### Cell cycle arrest

Carvacrol has been found to arrest the cell cycle at different checkpoints, preventing cancer cells from proliferating uncontrollably. By inhibiting cell cycle progression, carvacrol can halt the growth of cancer cells and impede tumor development.

#### Anti-proliferative effects

Carvacrol exhibits anti-proliferative properties by inhibiting the proliferation of cancer cells. It interferes with cellular processes involved in cell growth and division, thereby suppressing the proliferation of cancerous cells and reducing tumor size [162].

#### Inhibition of angiogenesis

Carvacrol has been shown to inhibit angiogenesis, the formation of new blood vessels that supply nutrients and oxygen to tumors. By disrupting angiogenesis, carvacrol deprives cancer cells of essential resources for their growth and metastasis, thereby impeding tumor progression.

#### Modulation of signaling pathways

Carvacrol can modulate various signaling pathways involved in cancer progression, including those related to cell proliferation, survival, and metastasis. By interfering with these pathways, carvacrol exerts control over cancer cell behavior and inhibits tumor growth and spread [162].

Overall, the diverse pharmacological properties of carvacrol, including its ability to induce apoptosis, arrest the cell cycle, inhibit proliferation, reduce inflammation, scavenge free radicals, inhibit angiogenesis, and modulate signaling pathways, collectively contribute to its potential as an anticancer agent [161, 163]. However, further research, particularly clinical studies, is needed to fully elucidate its efficacy, safety, and optimal therapeutic applications in cancer treatment [164].

## Conclusions

Over the past 25 years, around 65% of anticancer drugs have been derived from natural sources, with chemical synthesis enabling large-scale production and the creation of novel analogs. However, many natural compounds face challenges like low solubility and poor bioavailability. Nanotechnology addresses these issues by enhancing drug solubility, biocompatibility, and surface modification while enabling tumor targeting and multidrug resistance (MDR) management. NP-based drug delivery systems (NP-based DDS) offer improved pharmacokinetics, stability, and efficacy compared to traditional therapies, serving as platforms for combination treatments.

While combining phytochemicals with chemotherapy shows promising results, challenges remain in developing optimized nanocarriers and achieving synchronized pharmacokinetics at tumor sites. Though preclinical studies are encouraging, phyto-nanoformulations are not yet clinically viable. Risks related to NP safety and efficacy must also be addressed. Nonetheless, researchers believe phytochemical-based nanoformulations will soon become integral to cancer treatment, meeting international toxicity and biocompatibility standards.

## Abbreviations

AgNPs: Silver nanoparticles
BSA: bovine serum albumin
CTLs: cytotoxic T lymphocytes
ETP: etoposide
EXOs: exosomes
FA: folic acid
LNPs: liposome nanoparticles
MDR: multidrug resistance
MPA: mycophenolic acid
NMOFs: nanoscale metal-organic frameworks
NPs: nanoparticles
PACA: poly (alkyl cyanoacrylate)
PCL: poly-caprolactone
PDT: photodynamic therapy
PEG: polyethylene glycol
PLA: polylactic acid
PS: photosensitizer
PSQ: polysilsesquioxane
PTX: paclitaxel
QC: quercetin
QDs: quantum dots
SLN: solid lipid nanoparticles
VCR: vinca alkaloids vincristine

## Declarations

### Copyright

© The Author(s) 2025.
